# Atypical Radiographic Presentation of *Cryptococcus* Pneumonia in a Newly Diagnosed HIV Patient

**DOI:** 10.1155/2019/9032958

**Published:** 2019-03-27

**Authors:** Arthur Cacacho, Umair Ashraf, Arsalan Rehmani, Masooma Niazi, Misbahuddin Khaja

**Affiliations:** ^1^Division of Pulmonary and Critical Care Medicine, Bronx Care Health System, Icahn School of Medicine at Mount Sinai, 1650 Grand Concourse, Bronx, NY 10457, USA; ^2^Department of Medicine, Bronx Care Health System, Icahn School of Medicine at Mount Sinai, 1650 Grand Concourse, Bronx, NY 10457, USA; ^3^Division of Pathology, Bronx Care Health System, Icahn School of Medicine at Mount Sinai, 1650 Grand Concourse, Bronx, NY 10457, USA

## Abstract

**Background:**

*Cryptococcus* infection is an opportunistic infection that occurs primarily among immunocompromised patients, and the morbidity and mortality of this infection is high if left unrecognized and untreated. There are no clinical or radiographic characteristics typical of cryptococcal pneumonia, and its clinical and radiological presentations often overlap with other diagnoses.

**Case Presentation:**

We present a case of a 25-year-old man from Ghana admitted for an altered mental state, weight loss, neck pain, fever, and photophobia. He was diagnosed with *Cryptococcus neoformans* meningitis by cerebrospinal fluid culture and with disseminated cryptococcal infection by a positive *Cryptococcus* blood test. Diffuse micronodular opacities were found in a miliary pattern in the upper portions of both lungs upon imaging, which suggested miliary tuberculosis; thus, the patient was started on antituberculosis therapy. The patient underwent flexible fiber optic bronchoscopy, and transbronchial biopsy of the right lung showed bronchopneumonia with fungal spores consistent with filamentous *Cryptococcus neoformans*, which grew in tissue culture of the right lung. Interferon-gamma release assay, *Mycobacterium tuberculosis* PCR, and acid-fast bacilli staining of the bronchoalveolar lavage were negative for the *M. tuberculosis* complex.

**Conclusion:**

The similarities in clinical and imaging findings among patients with acute immunodeficiency syndrome with coinfections make diagnoses difficult; thus image-guided biopsies are essential to confirm diagnoses.

## 1. Introduction

*Cryptococcus* infection is an opportunistic infection that occurs primarily among people who are immunosuppressed. Cryptococcal meningitis is the most common presentation and causes significant morbidity and mortality [1], whereas infection of the lungs, skin, lymph nodes, and bones occur infrequently [2]. There are two main *Cryptococcus* species currently described: *Cryptococcus neoformans* and *Cryptococcus gattii*. Over the years as molecular techniques evolved, it has been shown that *Cryptococcus neoformans* and *Cryptococcus gattii* have many features of highly evolved species and have strain-specific differences that correspond to their geographic locations, environmental niches, host predilections, and clinical manifestations. Further insight into the pathobiology of these encapsulated yeasts, including their capacity to adapt to environmental pressures, exploit new geographic environments, and cause disease, is needed [3].

According to the U.S. Centers for Disease Control and Prevention, *Cryptococcus neoformans* is a major cause of illness in people living with human immunodeficiency virus (HIV)/acquired immunodeficiency syndrome (AIDS) with an estimated 220,000 cases of cryptococcal meningitis occurring worldwide each year with a case-fatality ratio of ∼12%. *Cryptococcus gattii* infections are rare, but the mortality rate ranges from 13 to 33% [4, 5]. Cryptococcal pneumonia has a variety of clinical manifestations and radiological appearances that are similar to other diseases. Although it is imperative to be aware of these presentations, diagnosis based purely on these features is difficult.

We present a case of a patient with atypical radiographic presentation of cryptococcal pneumonia documented by flexible fiberoptic bronchoscopy (FFB) with bronchoalveolar lavage (BAL) culture, transbronchial biopsy (TBBx), and lung tissue culture.

## 2. Case Presentation

A 25-year-old man from Ghana was admitted to our hospital for an altered mental state, weight loss, neck pain, cough, fever, nausea, vomiting, and photophobia for 7 days. He reported a cough with greenish phlegm and blood-tinged sputum and a weight loss of ∼20 lb in the last month. He reported that he could not eat because he was always nauseous and experienced nonbilious, nonprojectile vomiting. He denied chest pain, difficulty breathing and swallowing, diarrhea, dysuria, and skin rashes. His past medical history included intermittent asthma and migraines, but he was not on any medications. He did not have any significant family history or prior surgeries. He reported that he smoked 10 cigarettes a day for the past 5 years, drank alcohol occasionally, and denied illicit drug use except for marijuana. He reported that he last traveled to Ghana 6 months priorly, and he denied having contact with sick persons. He reported that he was sexually active with many partners but denied any prior sexually transmitted diseases.

Examination revealed a cachectic young male with a heart rate of 102 beats/min, a blood pressure of 108/69 mmHg, a respiratory rate of 20 breaths/min, a temperature of 98°F, and a 96% oxygen saturation in ambient air. He had a body mass index of 19.2 kg/m^2^ and was lethargic but oriented. He had no icterus or cervical or axillary lymphadenopathy. He had clear bilateral breath sounds, and his cardiovascular and abdominal examinations were unremarkable. He had a good pulse and no peripheral edema.

On admission, the patient tested positive for HIV with a CD4 count less than 20 and a high viral load. Pneumonia and meningitis resulting from opportunistic infections were considered, and he was treated for bacterial meningitis and tuberculosis (TB) even though the lumbar puncture was delayed because the patient was restless and uncooperative. The lumbar puncture results showed a pressure of 23 cm·H_2_O, a white blood cell count of 2, a red blood cell count of 57, a protein level of 102 mg/dl, and a glucose level of 25 mg/dl. The Gram stain and bacterial antigen tests were negative.

**Figure 1:**
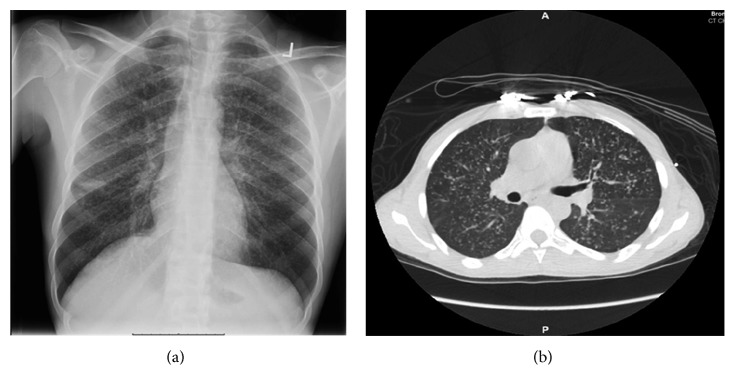
(a) Chest radiograph showing diffuse micronodules. (b) Axial view of the CT chest scan showing diffuse micronodules in a miliary pattern.

There was a high suspicion of pulmonary TB and tuberculous meningitis due to recent travel to Ghana, and the initial chest X-ray showed multiple nodular opacities suggestive of miliary TB (Figure 1(a)). The micronodular appearance was confirmed by a chest CT scan without contrast (Figure 1(b)). Despite a quantiFERON negative test result, quadruple therapy for TB with isoniazid, pyrazinamide, ethambutol, and rifampin (RIPE) was continued.

On succeeding days, yeast was growing in the patient's blood cultures and a Gram stain of the patient's cerebrospinal fluid (CSF) showed presence of yeast cells. The titer of *Cryptococcus* in the CSF was greater than 1 : 1024 and was eventually identified as *Cryptococcus neoformans*. Thus, antibacterial meningitis treatment was discontinued, and the patient was started on amphotericin B and flucytosine. Anti-TB medications were continued. He underwent placement of a lumbar drain due to a persistent headache and photophobia. Antiretroviral treatment was not started, but the patient was treated with prophylaxis medications for opportunistic infections. At this time, the patient had four persistently negative acid-fast bacilli sputum smears and was negative for the sputum *Mycobacterium tuberculosis* PCR test.

**Figure 2:**
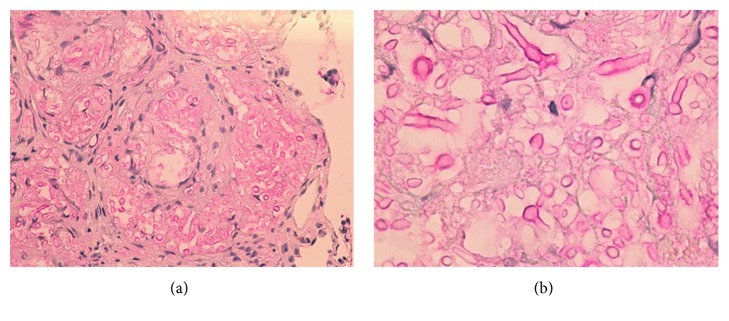
(a) Mucicarmine stain of the TBBx showing *C. neoformans* pleomorphic, single, and budding yeasts and filamentous pseudohyphae (400x magnification). (b) Mucicarmine stain of the TBBx showing *C. neoformans* yeasts and pseudohyphae with characteristic mucinous capsules (1000x magnification).

The patient underwent FFB with BAL and TBBx. In the BAL, >100,000 CFU/mL of *Staphylococcus haemolyticus* grew; thus, the treatment for bacterial pneumonia was modified. Tissue culture and TBBx showed the presence of *Cryptococcus neoformans*. Higher magnification microscopy showed the presence of both hyphae and yeast cells (Figures 2(a) and 2(b)). The patient showed clinical improvement and was discharged on oral fluconazole.

## 3. Discussion

Despite significant reductions in morbidity and mortality due to the availability of an effective combination of antiretroviral therapy, HIV infections still account for 1.5 million deaths annually [6]. Immunodeficiency increases the risk of opportunistic coinfections, which usually present as more severe, frequent, and atypical. Other noninfectious comorbid diseases are also of concern; thus, an immediate definitive diagnosis is essential to distinguish between diseases that coexist and diseases that mimic one other.

The five leading infectious diseases that continue to cause significant morbidity and mortality in HIV-infected individuals globally are TB, *Cryptococcus* infections, hepatitis B virus, hepatitis C virus, and malaria [6]. *Pneumocystis jiroveci *pneumonia is still a major concern; however, due to prophylaxis, its incidence has declined dramatically.

*Cryptococcus* infection remains the most significant and leading invasive fungal infection in the world today, and it accounts for an estimated 15% of all AIDS-related deaths globally. Humans likely become infected via inhalation. Although the factors that determine whether an exposed person develops symptomatic infection remain uncertain, one factor may include the size of the inoculum. The clinical manifestations of pulmonary cryptococcosis range from asymptomatic colonization of the airways to life-threatening pneumonia leading to acute respiratory distress syndrome [7]. Common symptoms include cough, sputum production, hemoptysis, dyspnea, chest pain, fever, malaise, night sweats, and weight loss [8]. The most common radiologic findings of pulmonary involvement include well-defined single or multiple noncalcified nodules, but pulmonary infiltrates, pleural effusions, hilar lymphadenopathy, and lung cavitation have also been observed [9].

Because the presentations of HIV coinfections sometimes overlap with their clinical presentations, diagnosis poses a significant challenge. Even radiologic imaging findings mimic other diseases, which increases the difficulty of diagnosis. Thus, image-guided biopsies are needed to confirm a diagnosis.

Because our patient had symptoms of pneumonia with radiographic findings highly suggestive of a miliary TB opportunistic infection, he was empirically treated with RIPE antituberculosis medication. These medications also provided coverage for atypical pneumonia, Gram-positive, and Gram-negative infections but did not cover cryptococcal pneumonia.

Radiographic features of pulmonary cryptococcosis are variable and have been described in many older case reports. In 1976, Hunt et al. published a case report which described cryptococcal pneumonia with multiple pulmonary nodules having central cavitation [10]. In 1980, Zlupko et al. reported a case of *Cryptococcus* with solitary and a few well-defined, noncalcified, pleural-based nodules [11]. In 1983, a case series of 23 patients by Feigin showed three different forms of radiopathologic findings including well-defined masses, segmental consolidation airway colonization with infiltrative masses, and colonization without parenchymal infiltrates [12].

Later studies described cryptococcal pneumonia among immunocompetent and immunocompromised patients. A retrospective evaluation of 10 immunocompetent patients showed that the most common findings were multiple, small (<10 mm in diameter) pulmonary nodules in the middle and upper lobes [13]. Pulmonary nodules were also observed in 12 patients with pulmonary cryptococcosis, the nodules were predominantly peripheral in distribution, and cavitation was observed in 40% of the cases [14]. Another retrospective study done on 17 immunocompetent patients with pneumococcal pneumonia showed that airspace consolidation was common, but that small, single and multiple nodules were rare in this group [15].

A study of 17 immunocompetent patients with pulmonary cryptococcosis by Shieh et al. in VGH-Taipei from 1967 to 1991 showed the following CXR findings: single nodule or mass (41.2%), multiple nodules (35.3%), and pneumonic patch or consolidation (23.5%). In addition, 29.4% had combined cavitary formation and 23.5% had pleural effusion [16]. Clinical analysis of 76 patients who were pathologically diagnosed with pulmonary cryptococcosis showed 85.53% were predominantly peripheral findings on chest CT scan (nodular masses 55.26%, infiltrates 23.68%, and mixed type 21.05%) [17].

The radiologic features of pulmonary cryptococcosis were also reviewed based on their CD4 T-lymphocyte levels. Nodules, masses, and halo signs were more common in the CD4 T-cell normal patients than on the patients with low CD4 T cell. The opposite trend was observed if there was presence of cavitations [18].

In a normal host, pulmonary nodules are the most common presentation. The nodules are characteristically subpleural and may be solitary or multiple with varying size. In disseminated disease, pulmonary nodules are still the most common manifestation, but in these cases, both cavitation and mediastinal lymphadenopathy are frequent. Diffuse reticulonodular interstitial opacities or miliary pattern may be present as well. In an immunocompromised patient who is HIV seropositive, the radiologic presentation may vary from normal findings to asymmetric interstitial, nodular, alveolar infiltrate, or miliary nodules [19].

Retrospective studies on HIV patients with cryptococcal pneumonia showed that interstitial infiltrates were common on chest X-rays [20, 21]. A more recent retrospective study among 43 immunocompromised patients with cryptococcal pneumonia showed pulmonary nodules with cavitation by chest computed tomography [22].

Thambidurai et al. published a case series in 2017 which showed that cryptococcal pneumonia had varied radiologic presentations. In one case, there was a disseminated miliary/reticulonodular pattern which was diagnosed as cryptococcal pneumonia after axillary lymph node biopsy. It was concluded that in immunocompromised patients with multiple pulmonary nodules, cryptococcal pneumonia, *Pneumocystis carinii* pneumonia, and mycobacterial infections should be considered in the differential diagnosis of pulmonary infections [23].

There is no clinical or radiographic feature unique to cryptococcal pneumonia. A high index of suspicion, especially among severely immunocompromised patients, is needed. Early diagnosis is essential for appropriate treatment.

## 4. Conclusion

In conclusion, to our knowledge, this is the first case report of an HIV patient diagnosed with cryptococcal pneumonia using FFB with TBBx. Chest radiography showed that the patient presented with diffuse and multiple small nodules in a miliary pattern, which is an unusual radiologic imaging finding that mimics other diseases, such as pulmonary TB; thus, early tissue diagnosis is essential for accurate diagnosis.

## Abbreviations

HIV: Human immunodeficiency virus
TB: Tuberculosis
BAL: Bronchoalveolar lavage.
